# TSP-1 Secreted by Bone Marrow Stromal Cells Contributes to Retinal Ganglion Cell Neurite Outgrowth and Survival

**DOI:** 10.1371/journal.pone.0002470

**Published:** 2008-06-25

**Authors:** Keming Yu, Jian Ge, James Bradley Summers, Fan Li, Xuan Liu, Ping Ma, Joseph Kaminski, Jing Zhuang

**Affiliations:** 1 State Key Laboratory of Ophthalmology, Zhongshan Ophthalmic Center, Sun Yat-sen University, Guangzhou, People's Republic of China; 2 Department of Radiology, University of South Alabama, Mobile, Alabama, United States of America; 3 Department of Radiology, Medical College of Georgia, Augusta, Georgia, United States of America; University of Washington, United States of America

## Abstract

**Background:**

Bone marrow stromal cells (BMSCs) are pluripotent and thereby a potential candidate for cell replacement therapy for central nervous system degenerative disorders and traumatic injury. However, the mechanism of their differentiation and effect on neural tissues has not been fully elucidated. This study evaluates the effect of BMSCs on neural cell growth and survival in a retinal ganglion cell (RGCs) model by assessing the effect of changes in the expression of a BMSC-secreted protein, thrombospondin-1 (TSP-1), as a putative mechanistic agent acting on RGCs.

**Methods and Findings:**

The effect of co-culturing BMSCs and RGCs *in vitro* was evaluated by measuring the following parameters: neurite outgrowth, RGC survival, BMSC neural-like differentiation, and the effect of TSP-1 on both cell lines under basal secretion conditions and when TSP-1 expression was inhibited. Our data show that BMSCs improved RGC survival and neurite outgrowth. Synaptophysin, MAP-2, and TGF-β expression are up-regulated in RGCs co-cultured with BMSCs. Interestingly, the BMSCs progressively displayed neural-like morphology over the seven-day study period. Restriction display polymerase chain reaction (RD-PCR) was performed to screen for differentially expressed genes in BMSCs cultured alone or co-cultured with RGCs. TSP-1, a multifactorial extracellular matrix protein, is critically important in the formation of neural connections during development, so its function in our co-culture model was investigated by small interfering RNA (siRNA) transfection. When TSP-1 expression was decreased with siRNA silencing, BMSCs had no impact on RGC survival, but reduced neurite outgrowth and decreased expression of synaptophysin, MAP-2 and TGF-β in RGCs. Furthermore, the number of BMSCs with neural-like characteristics was significantly decreased by more than two-fold using siRNA silencing.

**Conclusions:**

Our data suggest that the TSP-1 signaling pathway might have an important role in neural-like differentiation in BMSCs and neurite outgrowth in RGCs. This study provides new insights into the potential reparative mechanisms of neural cell repair.

## Introduction

BMSCs can self-renew,proliferate, and/or differentiate into a variety of cell types, such as cardiomyocytes, rhabdomyocytes, hepatocytes, osteocytes, chondrocytes, tencoytes, adipocytes, smooth muscle cells, and possibly even neural cells [1]–[6]. Therefore, BMSCs could play an important role in tissue repair by replacement of lost cells and stimulation of reparative processes within injured tissues [7]. For example, transplantation of BMSCs can improve functional recovery after stroke or other neurological trauma in animals [8]–[11]. Additionally, clinical trials with BMSCs are ongoing, and other clinical studies have been proposed to elucidate their usefulness [6], [12]–[16]. Koc and colleagues concluded that patients with Hurler syndrome and metachromatic leukodystrophy, both associated with skeletal muscle and neurological manifestations, might experience a reversal of disease pathology in some tissues treated with allogeneic BMSC infusion [13]. Additionally, Osiris, Inc. (Baltimore, MD) has been granted “Fast Track” designation from the United States Food and Drug Administration; patients who have undergone bone marrow transplantation and experiencing graft-versus-host disease are being enrolled into a Phase III trial [16].

Some researchers, including our group, have reported that BMSCs can differentiate into neural-like cells, with the expression of neural-associated proteins *in vivo* and *in vitro* [17]–[23]. For example, we reported that BMSCs can express NF and MAP-2 after treatment with all-trans retinoic acid, NF, and the schwann cell conditioned medium, or when co-cultured with neonatal rat retinal ganglion cells. However, the criteria for judging such transdifferentiation have been incomplete in many studies, often relying on genetic expression studies and morphological changes, rather than definitive proof of neuron-like characteristics such as synapse formation and appropriate electrophysiological properties. Conversely, several studies have demonstrated little or no neural differentiation *in vivo* after BMSC transplantation, although functional recovery appeared improved [24]–[29]. Still, based on additional observations, some investigators support the hypothesis that BMSCs secrete factors that promote neural recovery and survival. For example, BMSC treatment in an experimentally-induced ischemic stroke rat model facilitated axonal sprouting and remyelination in the cortical ischemic boundary zone and corpus callosum, which might have accounted for the neurological functional improvement observed [30], [31]. Intravenously administered BMSCs from male rats increased basic fibroblast growth factor expression, reduced apoptosis, promoted endogenous cellular proliferation, and improved functional recovery after ischemic stroke in female rats [32]–[34].

In order to elucidate the underlying reparative mechanisms, BMSCs and RGCs were co-cultured to evaluate for morphological changes. Additionally, RD-PCR was performed to detect differentially expressed BMSC genes when BMSCs were co-cultured with RGCs versus BMSCs cultured alone. Our data show that BMSCs and RGCs affect each other when co-cultured *in vitro*, resulting in BMSCs progressively displaying neural-like features over the seven-day study period, which is not likely achievable with simple chemical treatments [17]. Also, RGC survival and neurite outgrowth were improved. Synaptophysin, MAP-2 and TGF-β expression were up-regulated in RGCs co-cultured with BMSCs compared to RGCs cultured alone.

Interestingly, we found that TSP-1, an extracellular matrix glycoprotein with a variety of functions and best known for its antiangiogenic properties, was up-regulated in BMSCs co-cultured with RGCs compared to BMSCs cultured alone. TSP-1 is critically important in the formation of neural connections during development [35]-[37]. TSP-1 is highly expressed in the early postnatal rat cortex and superior colliculus during the period of intense synaptogenesis, but levels progressively decline as juvenile rats mature into adults. Immunocytochemical analysis revealed a 25–40% decrease in the density of synaptic puncta in the cortices of postnatal P8 and P21 mice lacking TSP-1 [38]. Therefore, we sought to investigate whether TSP-1 expression by BMSCs plays a role in neurite outgrowth of RGCs by co-culturing them with BMSCs. In order to evaluate the role of TSP-1, double siRNA transfection was used to transiently and selectively decrease endogenous expression of TSP-1. Silencing of TSP-1 with siRNA in BMSCs did not affect survival but reduced neurite outgrowth and expression of synaptophysin, MAP-2, and TGF-β in RGCs. Also, the number of neural-like BMSCs was significantly decreased by more than two-fold. Therefore, our data suggest that BMSCs and RGCs affect each other when co-cultured *in vitro*. The TSP-1 signaling pathway may play an important role in BMSC neural-like morphological change. We are unaware of any other study describing that BMSC-derived TSP-1 enhances RGC neurite formation, survival, and expression of synaptophysin, MAP-2, and TGF-β. Other researchers have shown that TSP-1 is involved in the healing process by suppressing inflammation which may result after an acute injury such as a wound or infarct. For example, Chatila and colleagues concluded that TSP-1 plays a critically important role after a myocardial infarct by limiting the expansion of fibrosis into the non-infarcted surrounding myocardium [39], while Uno and colleagues have suggested that TSP-1 accelerates corneal wound healing [40]–[42]. This study provides new insights on BMSC-mediated neuronal plasticity and the putative role of TSP-1 as a specific mediator of reparative processes in neurons.

## Materials and Methods

### Cell Culture

#### Primary BMSC culture

BMSCs were isolated by previously described methods. We utilized a well characterized technique that has been previously used by our lab (21, 22) and multiple others [17], [43].. Briefly, adult female Sprague Dawley rats (n = 5, 200–250 g body weight) were sacrificed by an intraperitoneal injection of Nembutal (60 mg/kg) (P3761, Sigma, St. Louis, MO). The tibias and femurs were extracted aseptically and rinsed with cold phosphate buffered saline (PBS, pH 7.4). A sterile 18-gauge needle was inserted into each bone marrow (BM) cavity, and the BM was extruded by injecting 10 ml of cold Dulbecco's Modified Eagle Medium (DMEM). The extruded BM was filtered through a 70-µm cell strainer (BD Biosciences, Bedford, MA) and centrifuged at 1200 rpm for 5 min. The BM pellet was resuspended in a flask containing DMEM, supplemented with 10% inactivated fetal bovine serum (FBS), 2 mM glutamine, 100 U/ml penicillin, and 100 mg/ml streptomycin. The culture medium was replaced to remove non-adherent cells after plating the cells for 24 hr. When the cells had grown to near confluency, passages of the cells were performed using 0.25% trypsin/1 mM EDTA. After 3–4 passages, the cells were stained with rabbit anti-rat IgG monoclonal antibodies CD44, CD71, CD106, and CD45 diluted 1∶100 (sc-9099, sc-25590, Santa Cruz Biotechnology, Santa Cruz, CA, BA0321, BA0406, Boster Biological Technology, Wuhan China). The BMSCs were used before the 6th passage, because they undergo morphological and functional changes after 10 passages that make them unsuitable for this type of experimentation.

#### Primary rat RGC cultures

RGCs were purified by sequential immunopanning to >99.5% purity from Sprague Dawley rats by previously described methods [44], [45]. Briefly, four 3-day-old Sprague Dawley rats were sacrificed by an intraperitoneal injection of Nembutal (P3761, 60 mg/kg) (Sigma, St. Louis, MO) and approximately 8 eyes were harvested for each experiment. The retinas that were separated from the enucleated eyeballs were incubated for 20 min in a solution containing 0.25% trypsin to dissociate the cells, followed by a 5 min incubation with mouse anti-macrophage monoclonal IgG diluted 1∶100 in antibody medium (sc-66204, Santa Cruz Biotechnology, Santa Cruz, CA). The cell suspensions were then incubated for 30 min on a 100-mm diameter petri dish coated with goat anti-mouse monoclonal IgG diluted 1∶100 in antibody medium (Santa Cruz Biotechnology, Santa Cruz, CA). Suspensions containing cells that did not adhere to the petri dish were collected and incubated for 1 hr on a 100-mm diameter petri dish coated with anti-Thy-1.1 monoclonal IgG diluted 1∶100 in antibody medium (sc-53116, Santa Cruz Biotechnology, Santa Cruz, CA). The cells that adhered to this pretri dish were released enzymatically (trypsin 0.125% for 10 min), after which they were cultured in 6-well plates coated with 0.1 mg/ml polylysine (P1399, Sigma, St. Louis, MO) in DMEM supplemented with 10% FBS and 25 uM glutamate (G0400, Sigma, St. Louis, MO) at 37°C in an atmosphere of 5% CO2 and 95% air with humidity. In order to assess the purity of RGCs, the cells were cultured in medium. After 3 days, FACS was performed to test the RGCs purity with a RGC specific antibody, FITC labelled Thy1.1. Briefly, 1×10^6^ cells/ml were harvested in PBS (10% FCS, 1% sodium azide), then incubated in 5 µg/ml of the FITC labeled Thy1.1 antibody (ab226, Abcam, Cambridge, MA ) for 30 minutes at room temperature. For the control, the cells were incubated with PBS. 2.5 mg/ml 7-AAD was added at this point for dead cell exclusion. The cells were washed three times by centrifugation at 400 rpm for 5 minutes and then resuspended in 500 ul ice cold PBS (10% FCS,1% sodium azide). FACS was performed to access the purity of RGCs.

#### Assay of BMSC and RGC survival and neurite outgrowth

2×10^5^ BMSCs and RGCs were co-cultured in a transwell system (0.45-uM pore size; BD Bioscience, Bedford, MA) in complete medium. After 5 days, the cover slips were fixed for 7 min in 4% paraformaldehyde, washed 3 times with PBS, and then incubated with 0.1% DAPI for 5 min. Following incubation, the cover slips were washed five times in PBS and mounted in Vectashield mounting medium on glass slides. Under fluorescent microscopy, 10 pictures were randomly taken from one slide in 10× microscopic fields. Cells with an intact nucleus were considered viable. For neurite outgrowth of RGCs, 10 pictures were taken randomly under a regular microscope from one slide. The neurite outgrowth of RGCs was assessed with a camera-lucida projection onto concentric circles. Outgrowth was determined by summing the lengths of all the cells and dividing by the total number of cells in 10× microscopic fields. The percentages of surviving and outgrowth of RGCs were determined on two or three cover slips for each experimental condition and normalized to the control cells examined in a similar manner. The average relative percent of surviving and outgrowth of RGCs from at least three separate experiments for each experimental condition is expressed in the text and figures as the mean+/−S.D.

#### RD-PCR assay

BMSCs and RGCs were co-cultured in complete medium in the transwell system described above. After 3 days, the total RNA, from the experimental and control conditions, respectively, was isolated with TRIzol Reagent (10296010, Invitrogen, Carlsbad, CA). RD-PCR was performed by previously described methods [46], [47]. Briefly, the cDNAs were synthesized with a cDNA kit (D2620, Takara, Dalian China). The cDNAs were digested at 37°C for 3 h in a mixture of 2 uL Sau3A1(10 U/uL ) (5′GATC3′) (D1082A, Takara, Dalian China) in total volume 20 uL of mixture. The two ends of each Sau3A1-digested fragment were linked to an adapter prepared by annealing the 2 oligonucleotides containing 5′-GATCCACACCAGCCAAACCCA-3′ (SIP) and 5′-GGTTTGGCTGGTGTG-3′ (SIR) in a ligation reaction containing 1 uL T4 DNA ligase (350 U/uL) (EL0011, Fermentas, Burlington Canada), 1 uL 10×DNA ligation buffer, 1 uL adapter (50 umol/L), and 20 pmol of *Sau*3A1-digested BMSC cDNA fragments; then, sterile water was added to make the final volume 10 uL. After 4 h of ligation at 16°C, PCR was performed in a 9700 thermocycler (Applied Biosystems, Foster City, CA) with the initial denaturation at 94°C for 5 min, followed by 35 cycles each at 94°C for 30 s, 60°C for 30 s, 72°C for 1 min, and a final extension at 72°C for 10 min. The PCR primers were designed to match the universal adapters, including the restriction site sequence, but with one “nesting” base overhanging the 3′-end. The reactions were divided into 10 subgroups [46], [47]. To evaluate the success of our RD-PCR, 7 uL of the PCR products were loaded onto 5% polyacrylamide gel to perform eletrophoresis at 90 V for 5–6 h followed by silver staining to separate different gene fragments.

The differentially expressed gene fragments or expressed sequence tags (EST) were isolated from the gel, and a second PCR was performed to investigate a band's contents. The PCR products were ligated with pMD-18-T Vector (D101C, Takara, Dalian China), and then sequenced with pMD-18-T sequencing primers. A BLAST search was performed on the EST sequences (http://www.ncbi.nlm.nih.gov/BLAST/Blast). The levels of mRNA and protein homologues for the EST were measured by semi-quantitative reverse transcriptase polymerase chain reaction (RT-PCR) and Western blot analysis, respectively.

#### Semi-quantitative RT-PCR and Western blot analysis

Total RNA was isolated with TRIzol Reagent. RT-PCR was performed using the one-step RT-PCR system (A1250, Promega, Madison, WI). The primer TSP-1 A (sense) is 5′GTTGCATGTGTGTGGAAGCAAC3′, and the primer TSP-1 B (antisense) is 5′ACCACACTGAAGATCTGGCCAG3′. The primer beta-actin A (sense) is 5′GATATCGCTGCGCTCGTCGTCG3′, and the primer beta-actin B (antisense) is 5′CGACGACGAGCGCAGCGATATC3′. For TSP-1, RT-PCR was performed for 35 cycles each at 94°C for 30 s, 60°C for 30 s, 72°C for 1 min, and a final extension at 72°C for 10 min. RT-PCR for beta-actin was performed for 20 cycles, each with the same temperature and time parameters as for TSP-1. Western blot analysis was performed by previously described methods [48]. The following IgG monoclonal antibodies were used: mouse anti-TSP-1 diluted 1∶1000 (BA24, Oncogene, San Diego, CA), rabbit anti-myc diluted 1∶1000 (sc-788, Santa Cruz Biotechnology, Santa Cruz, CA), and mouse anti-actin diluted 1∶1000 (sc-69879, Santa Cruz Biotechnology, Santa Cruz, CA). Proteins were visualized with horseradish peroxidase (HRP)-conjugated anti-rabbit, anti-mouse IgG diluted 1∶5000 (sc2004, Santa Cruz Biotechnology, sc2005, Santa Cruz, CA), followed by use of the ECL (HRP) Western blot analysis system (Santa Cruz Biotechnology, Santa Cruz, CA).

#### Measurement of TSP1 in co-culture medium by Western blot

1×10^6^ BMSCs and RGCs were co-cultured in a transwell system (0.45-uM pore size; BD Bioscience, Bedford, MA) in complete medium. After 24 hours, the medium was replaced with 10 ml of serum-free medium and the cells were incubated for 24 hours. The conditioned medium was collected, concentrated with a Centricon-10 (UFC910024, Billerica, MA), and protein concentrations were determined with the BCA assay (#23250, Pierce, Rockford, IL). Twenty µg of total protein was subjected to sodium dodecyl sulfate-polyacrylamide gel electrophoresis; after electrophoresis the proteins were transferred to a nitrocellulose membrane. Western blot analysis was performed by described methods above.

#### RNA interference (or silencing)

The siRNA sequences used for targeted silencing of TSP-1 expression and the control sequences were as follows: #TSP-1, GUUGGCACAAAGGCUCCA dTdT and #control, GGUUUGGCUGGGGUGUUAUdTdT. The oligos were purchased from Dharmacon (Lafayette, CO). Transfection of siRNA was performed using oligofectamine 2000 (11668-019, Invitrogen, Carlsbad, CA). Transfection was performed twice, 24 hours apart, to enhance the success of siRNA silencing. As a rescue experiment, some BMSCs were transduced at the second siRNA transfection with a recombinant adenovirus (pAd/CMV-Myc-TSP-1) expressing TSP-1, a fusion protein, and Myc-TSP-1. The TSP-1 cDNA sequence in the pAd/CMV-Myc-TSP-1 adenovirus expresses the same amino acids as BMSC-derived TSP-1 from rats but with different codons, thus these cDNA sequences are resistant to siRNA silencing. Expression levels of TSP-1 were measured by Western blot analysis on the third day

#### Effect of TSP-1 on BMSCs and RGCs

BMSCs were transfected with TSP-1 siRNA, and if rescued, they were then transduced with the TSP-1-expressing recombinant adenovirus as previously described. 48 hours after the first siRNA transfection, the cells were washed twice with sterile PBS. Trypsin buffer 0.125% was added to the petri dish and incubated at 37°C for 3 min. Trypsin digestion was inhibited by the addition of complete medium. Then, the BMSCs and RGCs were co-cultured in complete medium in the transwell system described above. After 3 and 5 days, an assessment of RGC survival and neurite outgrowth was performed as described above.

#### Synatophysin expression in RGCs

BMSCs were transfected with TSP-1 siRNA, and if rescued, they were then transduced with the TSP-1-expressing recombinant adenovirus as described previously. 48 hours after the first siRNA transfection, the cells were washed twice with sterile PBS. Trypsin buffer 0.125% was added to the petri dish and incubated at 37°C for 3 min. Trypsin digestion was inhibited by the addition of complete medium. The BMSCs and RGCs were then co-cultured in complete medium in the transwell system described above. After 3 days, RGCs were fixed for 7 min in 4% paraformaldehyde (PFA), washed three times in PBS, and blocked for 30 min in 100 uL of a blocking buffer containing 0.1% Triton X-100. After blocking, the cover slips were washed three times in PBS, and 100 uL of mouse anti-synaptophysin (BM0125, Boster Biological Technology, Wuhan China), diluted 1∶100 in antibody buffer, was added to each cover slip. For the control, the cover slips were incubated with PBS. The cover slips were then incubated overnight at 4°C, washed three times in PBS, and incubated for 2 hr with 100 uL of Cy3 conjugated sheep anti-mouse (BA1031, Bios, Beijing China), diluted 1∶50 in antibody buffer. Following incubation, the cover slips were washed five times in PBS and mounted in Vectashield mounting medium on glass slides. The pictures were taken by laser scan confocol microscopy (LSM 510 Meta, Zeiss, Germany). Western blot analysis was performed as described above.

#### Real-time RT-PCR

Total RNA was isolated with TRIzol Reagent (10296010, Invitrogen, Carlsbad, CA). One microgram of total RNA was subjected to reverse transcription with the SYBR PrimeScriptTM RT-PCR kit (DRR063S,Takara, Dalian China) following the manufactures protocol. Real-time PCR was employed to measure TSP-1, TSP-2, CD36, CD47, TGF-β, MAP-2, synaptophysin expression using the SYBR Green system (DRR063S,Takara, Dalian, China). The primers are displayed in Table 1. The thermal cycling conditions comprised an initial denaturation step at 95 °C for 3 min, then 40 cycles of two-step PCR including 95°C for 15 s and 60°C for 30 s. Data were collected during the 60°C. The amount of target gene mRNA relative to the internal control gene, β-actin, was calculated using the ΔCT method as follows: the relative expression = 2^−ΔCT^, ΔCT = C_T_(target gene)−C_T_(β-actin ) [49]. The data were analyzed in triplicate.

**Table 1:** The primers Real-time RT-PCR.

TSP-1	Sense	5′-GCCAGATGACAAGTTCCAAG-3′
	Antisense	5′-CTGACACCACTTGCTGCTTC-3′
TSP-2:	Sense	5′-AGTGGAGACAGGATGCTCTG-3′
	Antisense	5′-GGACGTAGTCAAACCGGACA-3′
CD36	Sense	5′-GTCTTCTATGTTCCAAACAC-3′
	Antisense	5′-TATAAACTGGATCTACAGTG-3′
CD47	Sense	5′-ACTCAAATACAAGTCCAGCC-3′
	Antisense	5′-CAAGACCAGAAGCGTTCTTC-3′
TGF-β	Sense	5′-TGCTTCAGCTCCACAGAGAA-3′
	Antisense	5′-TGGTTGTAGAGGGCAAGGAC-3′
Map-2	Sense	5′-ACCTGCCTGAGATGCTAGAT-3′
	Antisense	5′-TGTCATCAGCAACAGGTGGC-3′
Synaptophysin	Sense	5′- GTGTACTTTGATGCACCCTC -3′
	Antisense	5′- TCTGCAGGAAGATGTAGGTG -3′

#### Statistical Analysis

Data are expressed as means+/−SE. The differences between mean values were evaluated with the two-tailed Student's t-test (for 2 groups) and the analysis of variance (ANOVA, for >2 groups). All calculations and statistical tests were performed by the computer programs Microsoft Excel 2003 (Microsoft, Redmond, WA) or SPSS 11.5 (SPSS, Chicago, IL). p<0.05 was considered significant for all analyses.

## Results

### BMSC neural differentiation and their effect on RGCs survival and outgrowth

**Figure 1:**
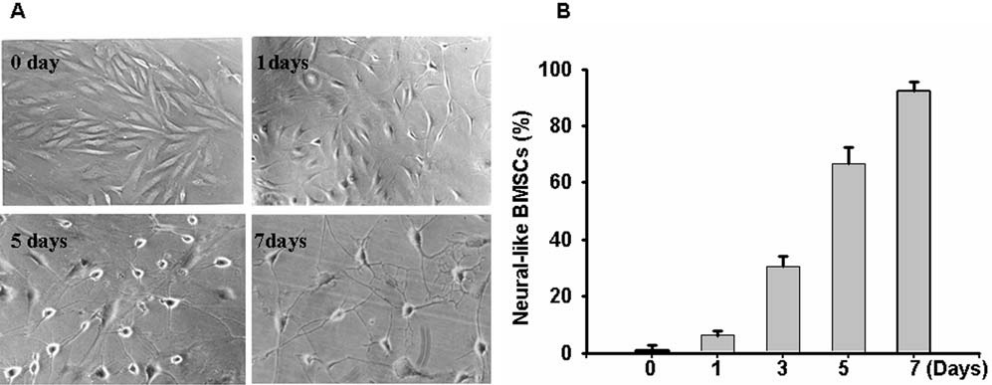
The morphology of the BMSCs after co-culturing. A, The morphological changes of BMSCs over 7 days (10×); B, The bar graph demonstrates that the percentage of neural-like BMSCs increases when co-cultured with RGCs. p≤0.05 at all time points. (10 pictures were taken randomly under a regular microscope from culturing cells at different time points. The average relative percent of neural-like BMSCs come from at least three separate experiments).

After BMSCs were co-cultured with RGCs, changes in BMSC morphology were observed after the first day (Figure 1A). Initially, the cytoplasm in the BMSCs retracted toward the nucleus and the cell bodies became more rounded. Cells continued along this progression with eventual neurite-like outgrowth, displaying primary and secondary branching. Over the subsequent 7 days, 92.2% (p = 0.026) of the BMSCs had a similar morphology (Figure 1B), while the remaining 7.8% of BMSCs had nonspecific morphological changes (p = 0.031). However, after 15 days of co-culturing with RGCs, BMSCs gradually died.

**Figure 2:**
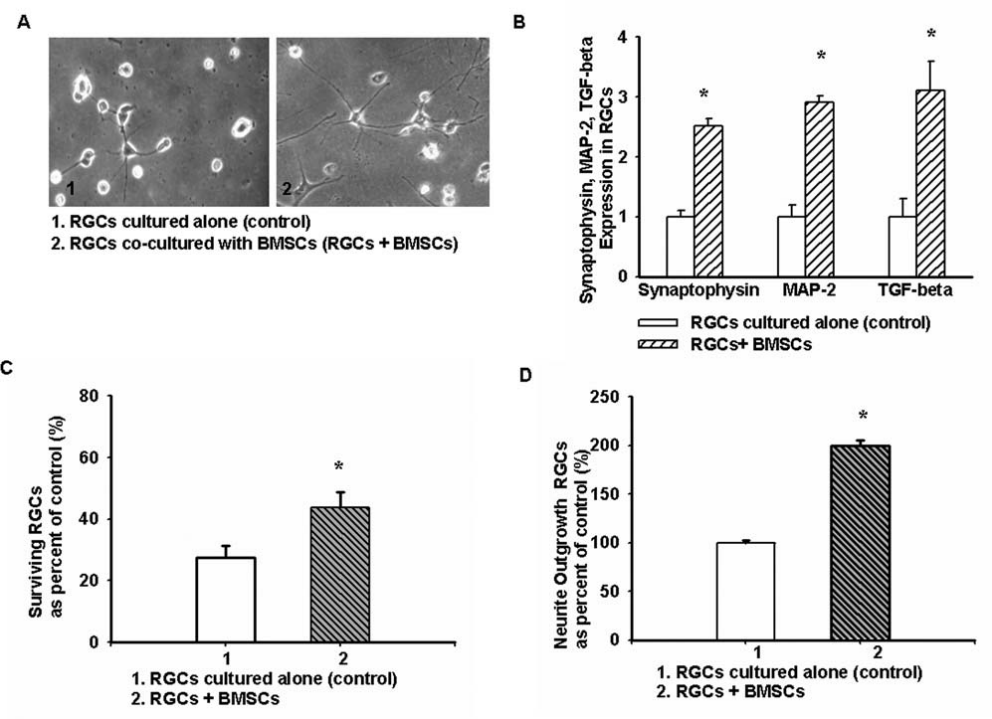
The effect of BMSCs on RGCs. A, RGC neurite formation was enhanced after 5 days of co-culturing with BMSCs (10×). B, Synaptophysin, MAP-2 and TGF-β expression were up-regulated in RGCs co-cultured with BMSCs as determined by real-time RT-PCR (p = 0.031, p = 0.023, p = 0.015, respectively). C, RGC survival was increased when co-cultured with BMSCs (p = 0.025) (d) The mean outgrowth of neurites in RGCs co-cultured with BMSCs increased (p = 0.03). *p-values≤0.05. (10 pictures were taken randomly under a regular microscope from culturing cells at different time points. The average relative percent of surviving and outgrowth of RGCs come from at least three separate experiments).

Phase contrast photographs ((Figure 2A) were taken from one well each containing RGCs cultured alone (control) and RGCs co-cultured with BMSCs (experimental group). BMSCs not only promoted RGC survival, but also significantly increased the formation of fine neurites with branching cones in RGCs compared to controls. According to our counting strategy, co-culturing of RGCs with BMSCs yielded a survival increase of up to 1.7-fold (Figure 2C), and more than 70% of RGCs had intact neurites after 5 days of co-culturing, compared to RGC controls with only 8.2% (p = 0.021). Figure 2D shows that BMSCs had a significant effect on neurite outgrowth of RGCs by increasing the number of primary and secondary branches. Real-time RT-PCR data showed that synaptophysin, MAP-2 and TGF-β expression were up-regulated in RGCs co-cultured with BMSCs as compared to RGCs cultured alone (Figure 2B).

### TSP-1 is up-regulated in BMSCs co-cultured with RGCs

**Figure 3:**
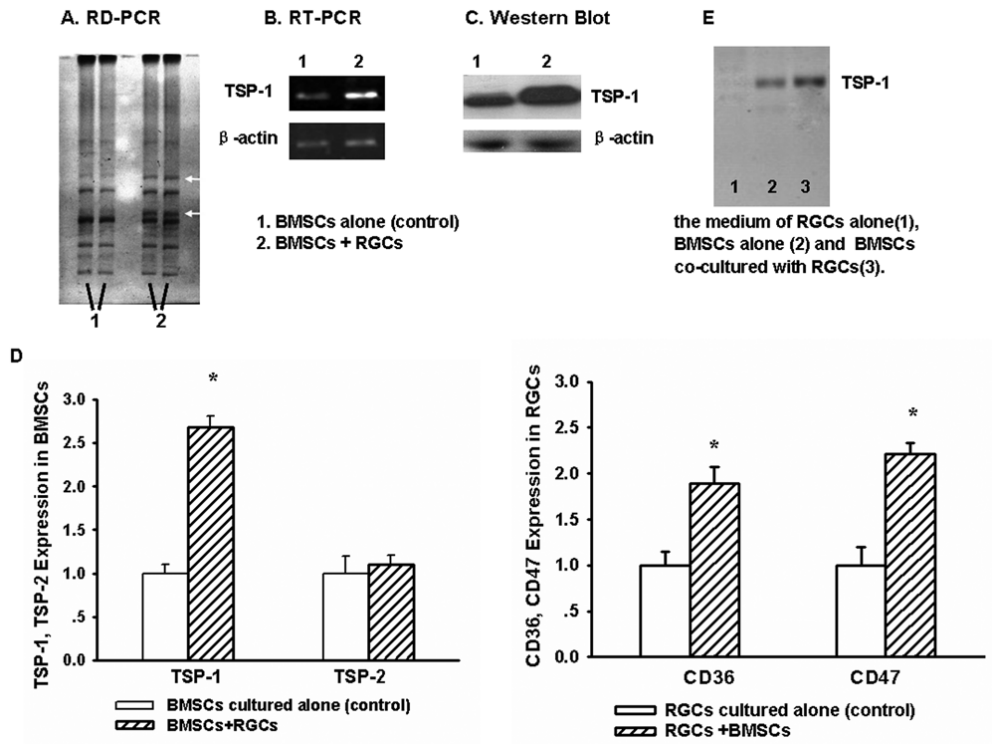
Patterns of differentially expressed genes in BMSCs. A, 5% polyacrylamide gel eletrophoresis demonstrates two dense bands representing upregulated genes in BMSCs co-cultured with RGC (white arrowheads). B, RT-PCR using total RNA isolated from BMSCs on day three demonstrated increased TSP-1 mRNA expression compared to controls. C, BMSC TSP-1 protein expression was increased as compared to the controls as demonstrated by Western blot. β-actin was used as a loading control in B and C. D, TSP-1, TSP-2 and TSP-1 receptors (CD36, CD47) expression were determined by real-time RT-PCR. E, Western analysis for TSP-1 in co-culture medium. *p-values≤0.05. (All data come from at least three separate experiments).

To assay mRNA expression profiles which might account for the observed morphological changes when BMSCs are co-cultured with RGCs, RD-PCR was performed with BMSCs cultured alone and with RGCs. The RD-PCR products were separated by eletrophoresis on 5% polyacrylamide gel and stained with a silver solution (Figure 3A). Two bands were noticeably denser compared to the controls. PCR was then performed with the fragments from the two bands purified from the gel as templates. After sequencing, one of the fragments was homologous with TSP-1 by a BLAST search online, and the other was homologous with another rat gene and will be the subject of future investigations. Our RT-PCR and Western blot data show that the TSP-1 gene is up-regulated when BMSCs are co-cultured with RGCs for 3 days (Figure 3B and 3C). Real-time RT-PCR data demonstrated that the expression of TSP-1 is up-regulated also (p = 0.037), but TSP-2 gene, another family member, is not changed, (Figure 3D). The expression of TSP-1 receptors (CD36, CD47) are up-regulated in RGCs (p = 0.012, p = 0.014, respectively) (Figure 3D). Additionally, the evidence of TSP-1 up-regulation is confirmed in co-cultured media by Western blot analysis (Figure 3E).

### siRNA mediated down-regulation of TSP-1 in BMSCs

**Figure 4:**
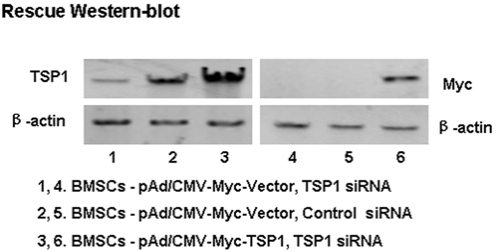
Endogenous TSP-1 expression was silenced by siRNA and rescued by infection with a recombinant adenovirus containing the wild-type TSP-1 cDNA driven by the CMV promoter (pAd/CMV-Myc-TSP-1). (The experiments were repeated at least three times).

TSP-1 is an extracellular matrix protein critically important in the formation of neural connections during development. TSP-1 is expressed at high levels in the early postnatal rat cortex and superior colliculus, during the period of intense synaptogenesis, but its expression is reduced in the adult. We sought to determine whether TSP-1 secreted by BMSCs affects neural cell survival and neurite outgrowth. In order to answer this question, siRNA interference was performed to silence TSP-1 expression. Transfection of BMSCs with siRNA oligos using a cationic lipid might affect expression of other proteins and reduce experimental reliability. Thus, we also used controls to overcome siRNA silencing of TSP-1 by transducing BMSCs with a recombinant adenovirus with a CMV-promoter containing TSP-1 cDNA (pAd/CMV-Myc-TSP-1). We used a two-time tandem RNA transfection approach at an interval of 1 day, then transduced BMSCs with the recombinant adenovirus (pAd/CMV-Myc-TSP-1) expressing TSP-1, a fusion protein, and Myc-TSP-1, coincident with the second siRNA transfection. The cells were harvested on the third day after the first transfection. This protocol produced a marked reduction in TSP-1 expression. However, TSP-1 expression was rescued by pAd/CMV-Myc-TSP-1 transduction (Figure 4, lane 3). Western blot confirmed exogenous TSP-1 expression, which was corroborated by antibody staining for Myc (Figure 4, lane 6). Of note, a start codon “ATG” is not present before the Myc epitope in the TSP-1 negative adenoviral vector, thus antibody stainings for Myc are negative in lanes 4 and 5.

### TSP-1 silencing significantly affects BMSC morphology and inhibits RGC neurite outgrowth, but does not alter BMSC and RGC survival

**Figure 5:**
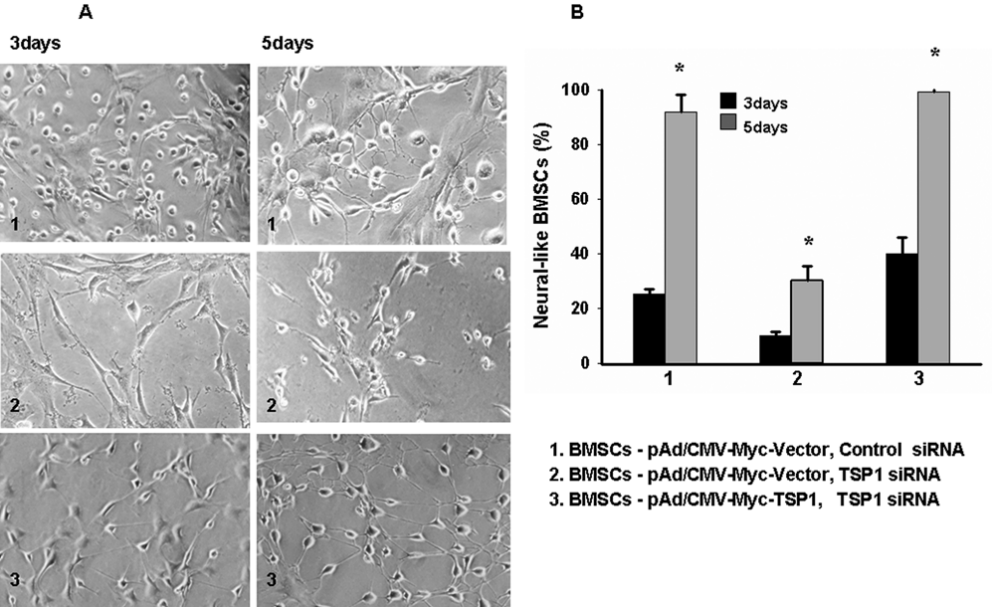
Morphological changes of BMSCs after co-culturing with RGCs when TSP-1 was silenced with siRNA and rescued by infection with a recombinant adenovirus expressing TPS1. 48 hours after the first siRNA transfection, BMSCs and RGCs were co-cultured in a transwell system. A, Neural-like morphological changes (10×) in BMSCs was increased at 3 and 5 days in controls (1). This effect was diminished when TSP-1 was silenced with siRNA (2), but restored in BMSCs infected with a TSP-1-expressing adenovirus (3). B, Bar graph shows the mean and SE of the percentage of neural-like cells in the control, silenced, and rescued BMSCs (p = 0.011, p = 0.022, p = 0.028, respectively). *p-values≤0.05. (10 pictures were taken randomly under a regular microscope from culturing cells at different time points. The average relative percent of neural-like BMSCs come from at least three separate experiments).

Some BMSCs were transfected with siRNA oligos and infected with a recombinant adenovirus (pAd/CMV-Myc-TSP-1) expressing TSP-1 before co-culturing them with RGCs as described above. Figure 5A shows that BMSCs with TSP-1 silencing with siRNA have fewer neural-like cells than the control BMSCs (i.e., without TSP-1 silencing) co-cultured with RGCs. The reduced number of neural-like cells is about 2.5-fold lower than control BMSCs ( p = 0.011). The morphology of BMSCs with siRNA silencing of TSP-1 was normalized to the controls infected with the recombinant adenovirus expressing TSP-1. Figure 5B shows quantification of neural-like cells observed with microscopy. After 15 days of co-culturing with RGCs, BMSCs gradually died. This data show that TSP-1 does not affect BMSC survival.

**Figure 6:**
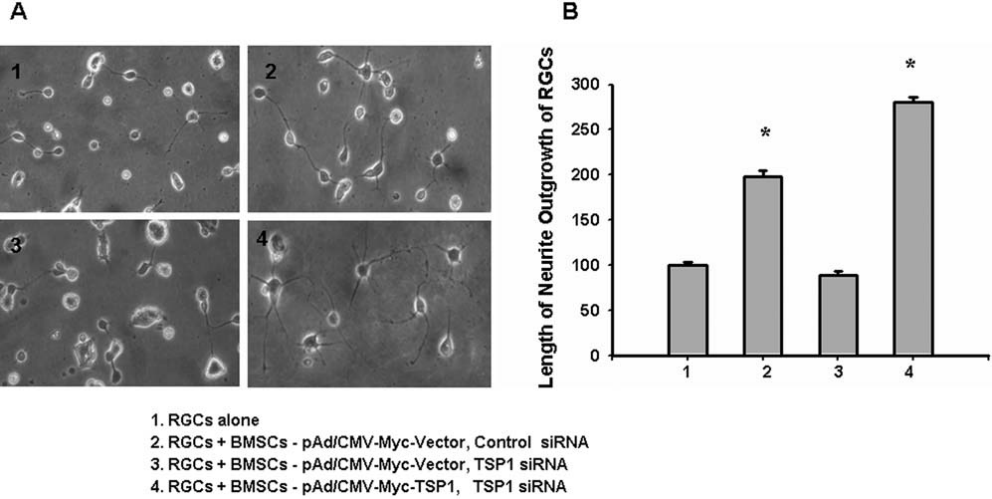
Morphological changes of RGCs after co-culturing with BMSCs with the endogenous TSP-1 silenced by siRNA and rescued by infection with a recombinant adenovirus expressing TSP-1. 48 hours after the first siRNA transfection, BMSCs and RGCs were co-cultured in a transwell system. A, After 5 days, RGC survival was not diminished by co-culturing with BMSCs with TSP-1 silencing, but the degree of RGC neurite outgrowth was lessened (3). B, Bar graph shows the mean and SE of RGC neurite outgrowth in within (10×) microscopic fields after 5 days of co-culturing. RGC neurite outgrowth was enhanced by co-culturing with BMSCs (2), which was diminished with TSP-1 silencing (3) but restored in TSP-1-rescued co-cultures (4) (p = 0.038). *p-values≤0.05. (10 pictures were taken randomly under a regular microscope from culturing cells at different time points. The average relative percent of outgrowth of RGCs come from at least three separate experiments).

Similarly, Figure 6A shows that BMSCs with TSP-1 silenced with siRNA do not alter RGC survival, but significantly inhibit RGC neurite outgrowth. Figure 6B shows quantification of RGC neurite formation observed with microscopy (10×) after 5 days of co-culturing.

### TSP-1 silencing affects the expression of synaptophysin, MAP-2 and TGF-β in RGCs

**Figure 7:**
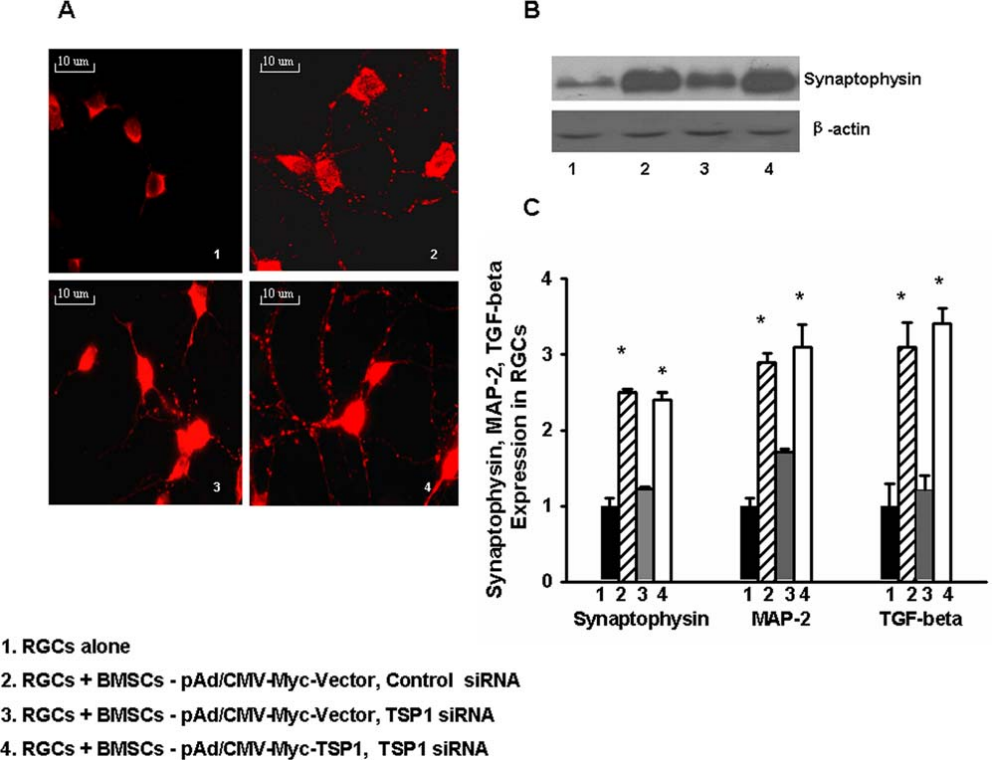
RGC synaptophysin, MAP-2 and TGF-β expression level were determined with and without BMSC (+/− TSP-1 siRNA silencing) co-culturing. BMSCs were transfected with siRNA oligos and infected with a recombinant adenovirus (pAd/CMV-Myc-TSP-1) expressing TSP-1 before co-culturing as described above. A, After 3 days, immunohistochemical analysis of RGCs for synaptophysin (red dots) shows more synaptic puncta when co-culturing with BMSCs compared to RGCs alone (2 & 4), but fewer when TSP-1 was silenced by siRNA (3). B, Western blot shows synaptophysin in RGCs is up-regulated by TSP-1; (2), which was diminished by TSP-1 silencing (3) and restored by TSP-1-expressing adenoviral rescue (4). C, After 3 days, synaptophysin, MAP-2 and TGF-β expression were determined by real-time RT-PCR (p = 0.019, p = 0.020, p = 0.017, respectively). *p-values≤0.05. (The experiments were repeated at least three times).

TSP-1 helps to promote normal CNS synaptogenesis in vivo and vitro [50], [51]. Synaptophysin is involved in the synapse formation of RGCs. In order to establish an assay to identify synaptogenic activities by BMSC-secreted TSP-1, the expression of synaptophysin in RGCs was assayed by immunohistochemistry and Western blot. Figure 7A, 7B show that the expression of synaptophysin in RGCs was up-regulated by co-culturing with BMSCs, and normalized when RGCs were co-cultured with BMSCs with endogenous TSP-1 silenced. RGC synaptophysin down-regulation was reversed when co-culturing with BMSCs (with TSP-1 siRNA) that were infected with the adenoviral vector expressing TSP-1. Additionally, TSP-1 affects MAP-2 and TGF-β, activated by TSP-1. Real-time RT-PCR data showed that synaptophysin, MAP-2 and TGF-β expression in RGCs were up-regulated by co-culturing with BMSCs, and normalized when RGCs were co-cultured with BMSCs with endogenous TSP-1 silenced (Figure 7C).

## Discussion

### The BMSCs and RGCs affect each other when co-cultured in vitro

BMSC differentiation into neural cells is a widely debated phenomenon [52], [53]. BMSCs are multipotent stem cells, which can differentiate into various cell types depending upon their local environment. When BMSCs were co-cultured with RGCs in vitro for over a week, the BMSCs differentiated into cells that exhibited some morphological characteristics of neural cell lineages, a result which is not likely achievable with simple chemical treatments. It is known that BMSCs can progressively assume neural-like morphological characteristics within the first 3 hours when treated with certain chemicals, such as DMSO, butylated hydroxyanisole, and isobutylmethylxanthine [17]. However, the mechanism underlying the induction of neural-like differentiation in BMSCs in RGC-conditioned medium in our experiments is likely different from that of chemical treatments because chemical treatments cause rapid morphological changes and cell death, whereas co-culturing causes a slower progression of morphological changes and delayed cell death.

Various factors secreted by RGCs might induce BMSC neural-like differentiation. For example, Mizobe and our group have reported that Thy-1.1, NF and MAP-2 expressed by RGCs accelerates neural-like differentiation in BMSCs [54]. Interestingly, we found in our study that the TSP-1 gene in BMSCs was up-regulated after co-culturing with RGCs. And real-time RT-PCR data showed that TSP-1 receptors (CD36, CD47) [55], [56] are up-regulated in RGCs co-cultured with BMSCs. TSP-1 may, in part, be responsible for the neurite-like outgrowth we observed in BMSCs. These observations suggest the possibility of using BMSCs as therapeutic agents in neurological diseases.

Furthermore, BMSCs significantly increased the propensity of RGCs to form neurites with branching growth cones and yielded an increased survival compared to controls. RGC survival increased by approximately 1.7 fold and more than 70% had intact neurites after 5 days of co-culturing with BMSCs, indicating that factors secreted by BMSCs may improve RGC survival and neurite outgrowth. And real-time RT-PCR data showed that synaptophysin, MAP-2 and TGF-β is up-regulated in RGCs co-cultured with BMSCs.

Therefore, BMSCs and RGCs seem to affect each other when co-cultured in vitro through increased expression of various proteins in both cell types. Likewise, the specific interactions leading to BMSC neural-like differentiation probably involve complex interactions with multiple proteins and/or signaling pathways that will need further elaboration.

### TSP-1 is up-regulated in BMSCs co-cultured with RGCs

Why is TSP-1 up-regulated in BMSCs co-cultured with RGCs? TSP-1 is secreted by a wide variety of epithelial and mesenchymal cells, in patterns that mirror developmental changes in the embryo and response to injury in the adult, and is critically important in the formation of neural connections during development [53], [54]. Brain macrophages contribute actively to neurite growth and regeneration during the development or in pathological states through the secretion of TSP [57]. After co-culturing with RGCs, the conditioned medium progressively induced neural-like differentiation in BMSCs. Initially, in the first day of co-culturing, the cytoplasm in one percent of BMSCs retracted toward the nucleus and began forming a rounded cell body. During such morphological changes, cell adhesion occurs in three distinct and identifiable stages: shrinking, spreading, and attaching. These stages involve interactions between integrins, along with accessory receptors such as syndecans, and their extracellular matrix substrates [58]. Therefore, TSP-1 likely plays a key role when BMSCs are induced by co-culturing with RGCs to differentiate into neural-like cells. This supposition is supported by our TSP-1 silencing and rescue experiments. These experiments showed that TSP-1 silencing reduced BMSC neural-like differentiation in RGC-conditioned medium; whereas, TSP-1 rescue in BMSCs with infection by a TSP-1-expressing adenovirus reversed the effect of siRNA silencing.

Thrombospondin family includes five members (TSP-1, TSP-2, TSP-3, TSP-4, and TSP-5)[51]. TSP-1 and TSP-2 are distinguished from the other three thrombospondins by a further set of three repeats, originally termed thrombospondin type 1 repeats. But our experimental data showed that only TSP-1 is up-regulated in BMSCs co-cultured with RGCs, TSP-2 is kept at the same level.

### TSP-1 secreted by BMSCs contributes to RGC neurite outgrowth and synaptogenesis

Thrombospondins are a family of multimeric, calcium-binding extracellular glycoproteins. The first identified thrombospondin, TSP-1, was discovered as a stored protein in a-granules that are released upon platelet activation. Initial studies of the function of TSP-1, therefore, focused on its role in platelet aggregation and fibrin clot formation, and also its effect on vascular endothelial and smooth muscle cells [59], [60]. In recent years, TSP-1 was found widely distributed in the embryonic extracellular matrix and expressed at high levels in the developing nervous system. Especially, in the postnatal but not adult CNS, TSP-1 is broadly localized in astrocytes and synapses, as expected for an astrocyte-secreted extracellular matrix molecule [61]. In addition, when peripheral nerves are damaged, TSP-1 expression is up-regulated and promotes neural growth and repair [36]. Therefore, the expression patterns and actions of TSP-1 in vitro suggest its potential role in the guidance of cell and growth cone migration. One report has shown that TSP-1 secreted by astrocytes is necessary and sufficient to induce synapse formation of ultrastructurally normal RGCs in vitro [38]. Mice lacking both TSP-1 genes have substantially fewer synapses in many CNS regions, indicating that TSP-1 helps promote normal CNS synaptogenesis in vivo [38]. Our RT-PCR and Western blot data show that TSP-1 is up-regulated in BMSCs co-cultured with RGCs. When TSP-1 expression was decreased with siRNA, BMSCs did not affect RGC survival, but rather reduced neurite outgrowth and the expression of synaptophysin in RGCs. Synaptophysin was the first cloned synaptic vesicle (SV) protein. Since its discovery in 1985 [62], synaptophysin has been used by many laboratories around the world as an invaluable marker to study the distribution of synapses in the brain and to uncover the basic features of the life cycle of SVs. Although single gene ablation of synaptophysin does not lead to an overt phenotype, a large body of experimental data, both in vitro and in vivo, indicate that synaptophysin (alone or in association with homologous proteins) is involved in multiple, important aspects of SV exo-endocytosis, including the regulation of SNARE (soluble N-ethylmaleimide-sensitive factor attachment protein receptor) assembly into the fusion core complex, formation of the fusion pore initiating neurotransmitter release, activation of SV endocytosis, and SV biogenesis [61], [63], [64]. Therefore, our results suggest that TSP-1 secreted by BMSCs plays a key role during synaptogenesis of RGCs in vitro.

How does TSP-1 induce RGC neurite outgrowth and synaptogenesis? Real-time RT-PCR data showed that TGF-β, activated by TSP-1, is up-regulated in RGCs co-cultured with BMSCs. TGF-β is a multifunctional cytokine with anti-inflammatory, reparative and neuroprotective functions [65]-[67]. Dhandapani and Brann demonstrated that the lack of TGF-β1 expression in neonatal TGF-β1 (−/−) mice results in a widespread increase in degenerating neurons accompanied by prominent microgliosis and reduced expression of synaptophysin and laminin [68]. Furthermore, TGF-β1 loss also strongly reduces survival of primary neurons cultured from TGF-β1 (−/−) mice. The authors suggest that TGF-β1 regulates the expression and ratio of apoptotic (Bad) and antiapoptotic proteins (Bcl-2, Bcl-×1), creating an environment favorable for cell survival of death-inducing insults [69]. This evidence strongly suggests the possibility that TSP-1 may act, in part, by inducing neurons to alter expression of proteins that participate in synaptogenesis.

In conclusion, we have shown that BMSCs improve RGC survival and neurite outgrowth. Meanwhile, the RGC-conditioned medium induces neural-like differentiation in BMSCs. During differentiation, TSP-1 in BMSCs is up-regulated after co-culturing with RGCs, and TSP-1 is probably involved in RGC neurite outgrowth and synaptogenesis. This study provides new insights into BMSC-mediated reparative mechanisms that might one day be translated into novel treatments for neurological disorders.
